# Reproducibility and feasibility of an online self-administered food frequency questionnaire for use among adult Norwegians

**DOI:** 10.29219/fnr.v65.7561

**Published:** 2021-11-10

**Authors:** Monica Hauger Carlsen, Lene F. Andersen, Anette Hjartåker

**Affiliations:** Department of Nutrition, Faculty of Medicine, Institute of Basic Medical Sciences, University of Oslo, Norway

**Keywords:** online dietary assessment, test-retest, nutrient intake, food intake, habitual diet, web-based FFQ, webFFQ

## Abstract

**Background:**

New methods of dietary assessment are increasingly making use of online technologies. The development of a new online food frequency questionnaire warranted investigation of its feasibility and the reproducibility of its results.

**Objective:**

To investigate the feasibility and reproducibility of a newly developed online FFQ (WebFFQ).

**Design:**

The semiquantitative WebFFQ was designed to assess the habitual diet the previous year, with questions about frequency of intake and portion sizes. Estimations of portion sizes include both pictures and household measures, depending on the type of food in question. In two independent cross-sectional studies conducted in 2015 and 2016, adults were recruited by post following random selection from the general population. In the first study, participants (*n* = 229) filled in the WebFFQ and answered questions about its feasibility, and in two subsequent focus group meetings, participants (*n* = 9) discussed and gave feedback about the feasibility of the WebFFQ. In the second study, the WebFFQ’s reproducibility was assessed by asking participants (*n* = 164) to fill it in on two separate occasions, 12 weeks apart. Moreover, in the second study, participants were offered personal dietary feedback, a monetary gift certificate, or both, as incentives to complete the study.

**Results:**

In the feasibility study, evaluation form results showed that participants raised issues regarding the estimation of portion size and the intake of seasonal foods as being particularly challenging; furthermore, in the focus group discussions, personal feedback on diet was perceived to be a more motivating factor than monetary reward. In the reproducibility study, total food intake was lower in the second WebFFQ; however, 63% of the food groups were not significantly different from those in the first WebFFQ. Correlations of food intake ranged from 0.62 to 0.90, >86% of the participants were classified into the same or adjacent quartiles, and misclassification ranged from 0 to 3%. Average energy intake was 3.5% lower (*p* = 0.001), fiber showed the least difference at 1.6% (*p* = 0.007), and sugar intake differed the most at −6.8% (borderline significant, *p* = 0.08). Percentage energy obtained from macronutrients did not differ significantly between the first and second WebFFQs.

**Conclusion:**

Our results suggest that at group level, the WebFFQ showed good reproducibility for the estimations of intake of food groups, energy, and nutrients. The feasibility of the WebFFQ is good; however, revisions to further improve portion size estimations should be included in future versions. The WebFFQ is considered suitable for dietary assessments for healthy adults in the Norwegian population.

## Popular scientific summary

- New dietary assessment methods are increasingly making use of online technologies.
- This study investigated the feasibility and reproducibility of a new online food frequency questionnaire (WebFFQ).
- Estimating portion sizes and intake of seasonal foods were regarded as challenging by the participants.
- Portion size pictures were regarded by some as helpful.
- Reproducibility was overall good.
- The WebFFQ was shown to adequately reproduce intakes of food and nutrients in a general adult Norwegian population.”

Self-report instruments are used frequently in research on nutrition. All methods used to assess long-term or short-term diet, either prospectively or retrospectively, have associated measurement errors (1,2). Self-report instruments assessing long-term retrospective intake challenge the subjects’ memory and their ability to take into account the variability of intake by day and season, or to estimate portion sizes and frequencies of intake (1). Over the past decade, traditional paper-based food frequency questionnaires (FFQs) have been replaced by online questionnaires (3, 4). Digital solutions may minimize some of the errors associated with paper FFQs, and missing values can be minimized in an online FFQ due to the use of automated pop-up reminders and mandatory questions. With online FFQs, the use of pictures of portion sizes may ease the cognitive task of choosing the right portion size and has the potential to reduce errors of inaccurate estimations of portion size. Computerized data capture also leads to considerable reduction in working load compared to paper FFQs as data are stored automatically.

The use of online computer technology does not obviate all limitations of an FFQ (4), however. All methods used to assess long-term or short-term diet, either prospectively or retrospectively, have associated measurement errors (1, 2). An online, self-administered, semiquantitative FFQ with portion size pictures, the WebFFQ, has been developed at the University of Oslo (UiO), Norway. The WebFFQ is based on earlier paper FFQs developed at UiO (6, 7) and was developed with the purpose of facilitating secure data capture, reducing manual data handling and missing values, and making the user experience more positive. The WebFFQ has been validated using doubly labeled water and multiple 24-h recalls (8). The aims of the present study were to evaluate the feasibility and the reproducibility of the WebFFQ with regard to nutrient and food intakes.

## Methods

### WebFFQ

To gain access to the WebFFQ, participants had to log in to the secure governmental login system (MinID) to ensure their secure identification. An informed consent was also obtained via the WebFFQ.

The WebFFQ was designed to assess habitual diet, food and nutrient intakes at group level, as well as to rank individuals according to their intakes. The WebFFQ consisted of 279 questions about the types, frequency of consumption, and portion sizes of food and beverages that participants had consumed the previous year. The questions were grouped according to the following main categories: bread; spread and butter/margarine; breakfast cereals; yoghurt; cold (including milk), warm, and alcoholic beverages; dinner meals; vegetables and legumes; fruits, berries, spices, nuts, and seeds; cakes and desserts; snacks; and dietary supplements. The WebFFQ included pictures of different portion sizes for food items, for which portion size may be difficult to estimate, such as composite dishes or when the food items do not come in natural units such as slices, spoons, or cups. Frequency of consumption of each food or beverage ranged from never to several times per month, week, or day. If participants did not fill in certain questions, an automated prompt would make them aware of this. All questions were mandatory. To reduce the burden of participants, categories of food or beverage (i.e. yoghurt) could be bypassed for products never consumed (skip-algorithms). Anthropometric and demographic questions were placed at the end of the questionnaire. The WebFFQ is designed for use on PCs, tablets, and mobile phones. Depending on which device the participants use, an adaptive web-design changes the screen appearance of the WebFFQ.

Data from the WebFFQ were saved in the secure storage facility TSD (Services for Sensitive Data) at UiO, and the dietary data transferred to the food and nutrient calculation system KBS, version 7.3, at the Department of Nutrition, UiO (9). Estimations of food and nutrient intakes were performed in KBS food composition database AE14. Database AE14 is an extended version of the official Norwegian Food Composition Table, version 2014.

### Feasibility study design and participants

**Figure 1:**
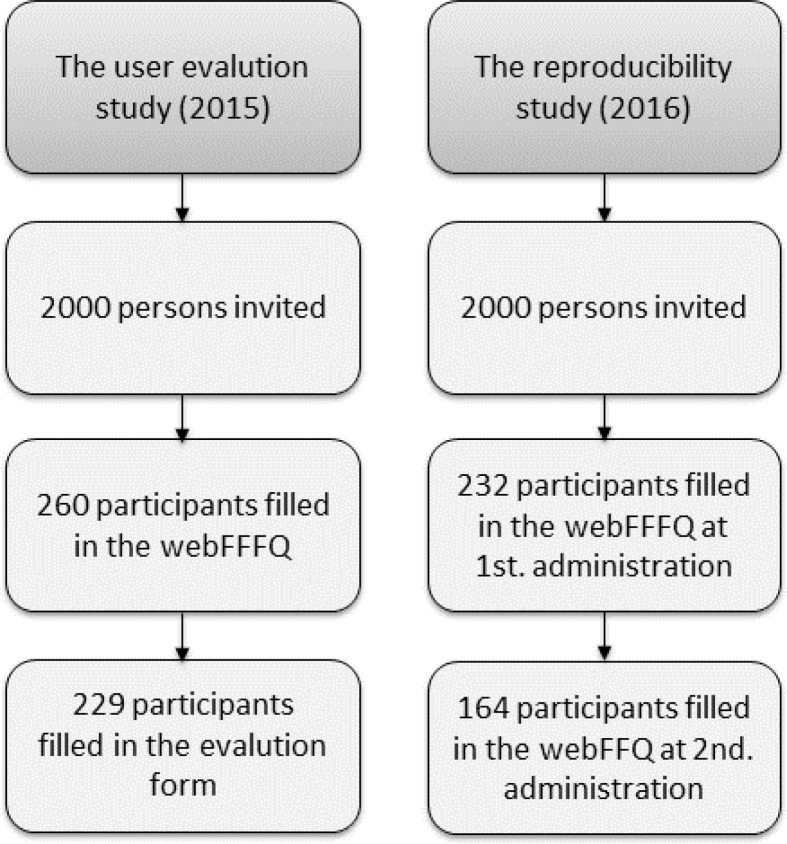
Overview of the two studies; the user evaluation study and the reproducibility study.

In January 2015, 2,000 adults between 18 and 75 years, selected randomly from the Norwegian National Population Registry, received invitations by post to take part in a study to evaluate the feasibility of the WebFFQ. The feasibility study included filling in the WebFFQ once and subsequently answering the feasibility evaluation form (Fig. 1), which was an additional web-based questionnaire with four questions about how participants experienced filling in the WebFFQ, including how much time they spent on it, whether they thought the WebFFQ was difficult or easy to fill in (five answer alternatives from ‘very easy’ to ‘very difficult’) and why, and whether there were any questions that were unclear. They were also asked whether they had any additional comments.

### Focus group meetings

In the feasibility evaluation form, participants were asked to indicate if they would like to be invited to focus group meetings. Those who indicated positively (*n* = 89) were invited via email. Two focus group meetings were organized a few weeks after the last participant had filled in the feasibility evaluation form, with three and six participants, respectively. The meetings were held at the Department of Nutrition, UiO, and conducted as semi-structured focus group interviews with one moderator and one assistant present in addition to the participants. During the focus group discussions, the participants were asked about the procedures of invitation and login; how they experienced the ease of use and structure of the WebFFQ; the use of incentives and reminders by SMS or telephone; the associated text with guidelines and information about the WebFFQ; and any other comments. The discussions were recorded, and the data were processed manually and summarized.

### Reproducibility study design and participants

One year later, in January 2016, another 2,000 individuals, selected randomly from the Norwegian National Population Registry, aged 18–75 years, were invited by post to participate in the reproducibility study. The invitation included information about how the study was organized, what the participants had to do, and the incentives offered to those who completed the study – personal written feedback about their diet and/or a 200 NOK gift certificate (approximately 20 €). The participants filled in the same WebFFQ twice, approximately 12 weeks apart, during January to March (first administration, WebFFQ1) and April to June (second administration, WebFFQ2) 2016 (Fig. 1).

The feasibility and reproducibility studies were conducted according to the Declaration of Helsinki, and a written informed consent was obtained from all participants. No data were collected about the persons who were invited but chose not to participate.

### Statistical analyses

Self-reported anthropometric data, estimated intakes of macronutrients, total energy, and percentage energy (E%) from all macronutrients except alcohol were normally distributed and presented as mean and standard deviation (SD) or 95% confidence intervals (CIs). Estimated intakes of alcohol, energy from alcohol, and intakes of food and micronutrients showed skewed distributions and are presented as median and 25th and 75th percentiles. One exception was the intake of vegetables, which was normally distributed. Paired-sample *t*-test and Wilcoxon signed-rank test were used to test for differences in intakes between WebFFQ1 and WebFFQ2, when data were normally and non-normally distributed, respectively. Correlations between estimates from WebFFQ1 and WebFFQ2 were performed using Pearson and Spearman correlations on normally and non-normally distributed data, respectively. For cross-classification analyses, intake estimates were ranked and classified into quartiles of intake. Misclassification was defined as classification into the opposite quartile. Differences in intakes estimated from WebFFQ1 and WebFFQ2, presented as absolute (g/day) and percentage, were calculated for each participant, and the group mean was calculated for each variable. Sample size calculations were based on a correlation coefficient of ≥0.5, with a significance level of 5% and a power of 90%, which resulted in a sample size of 76 (10). To estimate adequately the Bland–Altman limits for agreement between two methods, a sample size of 50–100 is required (11). Previous evaluation studies showed that we had to invite ~2,000 persons initially to obtain a group of 50–150 participants (11–13). Significance level was set at *p* < 0.05.

## Results

Table 1 presents demographic and lifestyle characteristics for the participants in the feasibility and reproducibility studies, respectively.

**Table 1:** Anthropometric and demographic data of the participants in the feasibility study and the reproducibility study

	The feasibility study						The reproducibility study					
	All, n=260		Men, n=112		Women, n=148		All, n=164		Men, n=68		Women, n=96	
	Mean	SD	Mean	SD	Mean	SD	Mean	SD	Mean	SD	Mean	SD
Age, years	46.2	16.4	46.7	17.5	45.9	15.5	46.3	15.5	48.2	15.1	45.0	15.6
Height, cm	172.6	9.3	180.4	6.3	166.7	6.3	173.3	9.2	181.4	6.8	167.5	5.7
Weight, kg	75.2	14.8	83.0	12.8	69.3	13.5	75.4	16.6	86.2	14.2	67.8	13.7
BMI, kg/m^2^	25.2	4.3	25.5	3.5	24.9	4.7	25.0	4.3	26.2	3.9	24.1	4.5
	Percent						Percent					
Age groups, years												
18-19, years	5.8		6.3		5.4		3.6		2.9		4.2	
20-44, years	37.3		35.7		38.5		41.5		35.3		45.8	
45-66, years	44.2		41.1		46.6		45.7		54.4		39.6	
67-75, years	12.7		16.9		9.5		9.1		7.4		10.4	
Non-smoking	89.6		87.5		91.2		89.6		94.1		86.5	
Married	49.2		50.9		48.0		48.8		50.0		47.9	
Cohabitant	20.4		19.6		20.9		24.4		20.6		27.1	
Living alone	23.8		24.1		23.7		23.2		23.5		23.9	
Other living arrangements	6.5		5.4		7.4		3.6		5.9		1.0	
Highest level of education:												
Primary school	6.2		3.6		8.1		2.4		0.0		4.2	
High school	37.3		42.9		33.1		39.6		42.6		37.5	
College, University	56.5		53.5		58.8		57.9		57.4		58.4	

### Feasibility study

During March and April 2015, 260 participants (13% of those invited) filled in the WebFFQ. The study population consisted of 112 men and 148 women, mean age 46.2 years (range 18–75), the mean BMI was 25.2 kg/m^2^, and 10% were smokers. More than 50% had completed higher education, and 70% were married or cohabiting (Table 1). Of the 260 participants, 229 also filled in the evaluation form (88%). Of these, 81% thought it was ‘very easy’ or ‘quite easy’ to fill in the WebFFQ, while 16% found it ‘a little difficult’ (Table 2). Mean time used filling in the WebFFQ was 38 minutes (95% CI: 36–41).

**Table 2:** Evaluation form answers, in the feasibility study, n= 229

Evaluation form question: “On a scale from ‘very easy’ to ‘very difficult’, how did you find the filling in of the WebFFQ to be?”		
Answer alternatives:	Number of participants, n (%)	Minutes used filling in the WebFFQ, mean (SD)
Very easy	51 (22)	34 (14)
Quite easy	136 (59)	39 (17)
A little difficult	37 (16)	40 (17)
Quite difficult	3 (1)	50 (35)
Very difficult	2 (1)	60 (42)

As many as 70% (*n* = 156) did not answer the question ‘Were there any unclear questions in the WebFFQ?’, 10% (*n* = 29) answered ‘no’ and 20% (*n* = 44) answered ‘yes’. The following issues were registered by participants who answered ‘yes’, in descending order: estimation of portion sizes; estimation of consumption frequency for foods associated with season; the WebFFQ was too long; filling in the WebFFQ took too much time; and the list of foods and beverages had too few alternatives for those on diets or who were vegetarians.

Twenty-one participants (9%) made positive comments; the pictures were helpful in estimating portion sizes (3%, *n* = 7), and the WebFFQ was easy to understand and use (6%, *n* = 14). The majority of the participants who made positive comments found the WebFFQ to be either ‘very easy’ or ‘quite easy’ to fill in (*n* = 19), while two of them found it ‘a little difficult’.

### Focus group results

Nine of the 89 invited participants had the opportunity and time to attend the focus group meetings as planned. The ages of the focus group participants ranged from 20 to 64 years. The following points regarding study design were underlined as important by the focus group participants: invitation by post was preferred over e-mail. An invitation letter by post was perceived to be more formal and serious than one sent by e-mail. Using MinID as the login system was perceived to be positive by eight of the nine participants. The focus group participants thought that an economic incentive would not have increased their motivation to participate; however, receipt of personalized dietary feedback after completing the study was viewed as motivating. Several of the focus group participants would have preferred easier access to written guidelines at every step in the WebFFQ. With regard to reminders, one reminder by SMS or telephone was perceived to be positive, a helping hand for those who ‘had just forgotten to do it’. However, the sending of more than one reminder was seen as inappropriate and unnecessary.

### Reproducibility study

Of the 2,000 people invited to partake in the reproducibility study, 232 (11.6%) gave written consent and filled in the WebFFQ once (WebFFQ1). After 3 months, 71% of the 232 participants filled in the WebFFQ a second time (WebFFQ2). Thus, the final study population consisted of 164 participants, of whom 59% were women (Fig. 1) and reflected, therefore, only a small proportion of the total invited sample. At group level, the women were of normal weight and 13.5% were smokers (Table 1). The male participants at group level were slightly overweight and 5.9% were smokers. The age range was 18–74 years for both sexes. Social status showed similar distributions for men and women, and the study population had a high share of participants with a high level of education (Table 1).

### Intake of food groups

Estimated intake of food groups is presented in Table 3 and showed no significant difference between first and second administrations of the WebFFQ for 15 of 24 food groups. In food groups with significant differences, the median differences in intake ranged from 0% for wine to 16% and 17% for fish and chocolate, respectively. Median differences of zero were observed for 10 food categories (Table 3). There was a general tendency toward lower intake of food and beverages in the second administration of the WebFFQ. Bland–Altman (BA) plot analyses showed large individual variations, with increasing differences, both positive and negative, with increasing mean intakes. All BA plot analyses of food groups showed the same patterns, and BA plots for intakes of bread, vegetables, red meat, and fish are presented in Appendix Fig. 1A–1D. Spearman correlation coefficients ranged from 0.62 for intake of bread to 0.90 for intake of coffee (Table 3). Cross-classification of participants into quartiles of intake showed that in all food groups, >50% of the participants were classified into the same quartile, and >86% were classified into the same or an adjacent quartile. Misclassification into the opposite quartile ranged from 0% for intakes of pasta, fruit and berries, fish and margarine, butter, and oils, to 3% for bread and milk (Table 3).

**Table 3:** Estimated intakes of food groups from the first (WebFFQ1) and second (WebFFQ2) administration of the WebFFQ, in the reproducibility study, n=164

Food group	WebFFQ1, g/d	WebFFQ2, g/d	p^a^	Difference, %^b^	rho^c^	Cross-classification		
	Median (IQR)	Median (IQR)		Median (IQR)		exact	exact + adj.	Miscl.^d^
**Bread**	**155 (83 to 222)**	**126 (72 to 209)**	**0.13**	**2.6 (-23.7 to 31.4)**	**0.62**	**52**	**86**	**3**
**Rice**	**16 (11 to 32)**	**16 (5 to 32)**	**0.12**	**0.0 (-50 to 50)**	**0.72**	**54**	**89**	**1**
**Pasta**	**22 (4 to 42)**	**16 (7 to 31)**	**0.04**	**2.5 (-23.4 to 48.3)**	**0.80**	**60**	**95**	**0**
**Cakes, buns and cookies**	**15 (6 to 29)**	**13 (6 to 27)**	**0.94**	**2.8 (42.9 to 34.1)**	**0.80**	**58**	**93**	**1**
**Potato and pot. products**	**52 (28 to 101)**	**51 (26 to 80)**	**<0.01**	**6.9 (-21.5 to 36.7)**	**0.78**	**60**	**91**	**1**
**Vegetables** ^e^	**424 (383, 465)**	**405 (365, 445)**	**0.18**	**7.8 (-19.8, 24.9)**	**0.76**	**55**	**93**	**1**
**Fruit and berries**	**180 (92 to 321)**	**160 (87 to 297)**	**0.05**	**7.7 (-25.5 to 31.1)**	**0.79**	**57**	**94**	**0**
**Juice**	**61(10 to 169)**	**61 (9 to 168)**	**0.87**	**0.0 (-64.1 to 50.0)**	**0.79**	**58**	**95**	**1**
**Meat, red**	**78 (52 to 127)**	**75 (45 to 115)**	**<0.01**	**10 (-17.8 to 30.8)**	**0.79**	**52**	**95**	**2**
**Meat, white**	**39 (22 to 65)**	**36 (18 to 57)**	**0.02**	**7.0 (26.1 to 40.1)**	**0.77**	**53**	**92**	**1**
**Fish**	**89 (58 to 129)**	**75 (47 to 117)**	**<0.01**	**16.3 (-18.4 to 35.1)**	**0.81**	**58**	**94**	**0**
**Egg**	**21 (13 to 38)**	**20 (13 to 38)**	**0.74**	**0.0 (-33.3 to 30.1)**	**0.75**	**58**	**91**	**1**
**Milk**	**167 (45 to 331)**	**158 (41 to 326)**	**0.04**	**3.5 (-38.3 to 39.0)**	**0.76**	**60**	**90**	**3**
**Yoghurt**	**34 (4 to 75)**	**28 (2 to 78)**	**0.61**	**0.0 (-48.4 to 50.0)**	**0.76**	**61**	**93**	**2**
**Cheese**	**26 (14 to 45)**	**26 (15 to 43)**	**0.93**	**0.6 (-51.5 to 32.9)**	**0.68**	**52**	**87**	**1**
**Margarine, butter, oils**	**24 (14 to 41)**	**24 (12 to 47)**	**0.72**	**4.3 (-43.8 to 31.4)**	**0.76**	**54**	**93**	**0**
**Chocolate**	**7 (2 to 15)**	**5 (1 to 12)**	**<0.01**	**17.3 (-33.7 to 68.6)**	**0.67**	**56**	**90**	**2**
**Coffee**	**451 (137 to 886)**	**420 (150 to 888)**	**0.23**	**0.0 (-6.0 to 25.9)**	**0.90**	**70**	**97**	**1**
**Soft drinks with sugar**	**8 (2 to 40)**	**11 (2 to 48)**	**0.11**	**0.0 (-167 to 62.4)**	**0.73**	**54**	**90**	**1**
**Soft drinks without sugar**	**7 (0 to 143)**	**7 (0 to 85)**	**0.36**	**0.0 (-10.1 to 78.9)**	**0.77**	**59**	**91**	**1**
**Tea**	**71 (9 to 361)**	**41 (7 to 375)**	**0.16**	**6.5 (-35.9 to 56.1)**	**0.86**	**58**	**95**	**1**
**Beer**	**36 (5 to 143)**	**36 (5 to 143)**	**0.59**	**0.0 (-13.9 to 20.1)**	**0.88**	**69**	**97**	**2**
**Wine**	**15 (2 to 41)**	**9 (2 to 37)**	**0.05**	**0.0 (-5.9 to 59.5)**	**0.88**	**65**	**96**	**1**
**All alcoholic beverages**	**63 (15 to 164)**	**57 (13 to 167)**	**0.47**	**0.0 (-24.5 to 27.0)**	**0.88**	**67**	**95**	**1**

a p-value, test of difference in intake between first and second WebFFQ, Wilcox.sign rank test. Significant difference set at p < 0.05.

b Percentage difference in intake between first and second WebFFQ, WebFFQ1-WebFFQ2.

c Spearman correlation between first and second WebFFQ.

d Misclassification of intake defined as opposite quartiles.

e Normal distribution, data and analyses presented with mean, 95%CI, t-test and Pearson correlation. FFQ, Food frequency questionnaire; g/d, gram per day; IQR, inter quartile range; 95% CI, 95% confidence interval.

### Intake of energy and macronutrients

Estimated intakes of energy and energy providing nutrients for all participants are presented in Table 4. Total energy intake estimated from WebFFQ2 was on average reduced by 0.81 MJ/day as compared with the estimate from WebFFQ1 (*p* = 0.001). The mean difference in energy intake at group level was 3.5% (Table 4). Intake of sugar, alcohol, and omega 3 fatty acids was not significantly different between the two time points. However, the absolute intakes of the other energy-providing nutrients were significantly different between the two time points, and the differences ranged from 1.4 g/day for polyunsaturated fatty acids (*p* = 0.03) to 18.7 g/day for carbohydrates (*p* = 0.002). Fiber showed the least difference, with 1.6% (*p* = 0.007), and sugar intake differed the most with −6.8%, however, borderline significant (*p* = 0.08) between the first and second administrations of the WebFFQ (Table 4).

**Table 4:** Estimated intakes of energy and energy providing nutrients from the first (WebFFQ1) and second (WebFFQ2) administration of the WebFFQ, in The Reproducibility Study, n=164

	WebFFQ1		WebFFQ2			p^a^	Difference, %^b^		r^c^	Cross-classification		
	Mean	95% CI	Mean		95% CI		Mean	95% CI		exact	exact + adj.	Miscl.^d^
**Absolute intakes**												
Energy, MJ/day	11.0	10.4, 11.7	10.2		9.6, 10.8	0.001	3.5	-1.2, 8.2	0.74	56	90	1
Protein, g/day	114	107, 121	104		98, 109	<0.001	5.5	1.4, 9.6	0.74	55	94	1
Fat, g/day	112	104, 120	105		97, 112	0.02	2.7	-2.7, 8.2	0.74	56	93	1
Saturated fat, g/day	37	34, 39	34		32, 37	0.02	2.1	-3.7, 7.9	0.67	54	92	0
Mono-unsat. fat, g/day	43	39, 46	40		37, 43	0.02	2.3	-3.4, 7.9	0.79	59	93	1
Poly-unsat. fat, g/day	22	20, 24	21		19, 23	0.03	1.9	-4.3, 8.3	0.79	60	93	1
Omega 3, g/day	7	6, 8	7		6, 8	0.06	2.3	-3.6, 8.2	0.90	59	96	0
Carbohydrates, g/day	263	246, 280	244		228, 260	0.002	1.8	-3.7, 7.3	0.75	56	93	1
Sugar, g/day	33	29, 37	30		27, 33	0.08	-6.8	-17.4, 3.7	0.66	59	93	3
Fiber, g/day	36	33, 39	34		31, 36	0.007	1.6	-4.4, 7.6	0.78	51	88	1
Alcohol, g/day^e^	5	1 to 9	4		1 –to 9	0.14	-5.7	-16.0, 4.7	0.89	68	96	0
**Energy percent, E%**												
Protein	17.9	17.4, 18.4	17.6	17.1, 18.1		0.10	5.5	1.4, 9.6	0.75	47	92	1
Fat	37.0	35.7, 38.2	37.2	35.9, 38.6		0.51	2.7	-2.7, 8.2	0.84	52	95	1
Carbohydrates	40.5	39.3, 41.8	40.7	39.4, 41.9		0.74	1.8	-3.7, 7.3	0.82	52	94	1
Sugar	4.9	4.4, 5.4	5.0	4.5, 5.5		0.63	-6.8	-17.4, 3.7	0.75	60	95	2
Fiber	2.7	2.5, 2.8	2.7	2.5, 2.8		0.59	1.6	-4.4, 7.6	0.86	61	92	0
Alcohol	1.4	0.3, 2.7	1.2	0.3, 2.7		0.65	-5.7	-16.0, 4.7	0.88	67	96	0

a p-value, test of difference in intake between first and second WebFFQ, Paired sample t-test. Significant difference set at p < 0.05.

b Percentage difference in intake between first and second WebFFQ, WebFFQ1-WebFFQ2.

c Pearson correlation between first and second WebFFQ.

d Misclassification of intake defined as opposite quartiles.

e Skewed distribution, data and analyses presented as median, Inter quartile range, Wilcoxon signed rank test and Spearman correlation. FFQ, Food frequency questionnaire; g/d, gram per day; 95% CI, 95% confidence interval.

Correlations between absolute intakes of macronutrients from the first and second administrations ranged from 0.66 for sugar to 0.90 for omega 3 fatty acids (all correlations significant at 0.01 level). Eight of 10 macronutrients, in addition to alcohol, showed high correlation (>0.7). Correct classification ranged from 51% for fiber to 68% for alcohol. Misclassifications into opposite quartiles were low for the absolute intakes of macronutrients (Table 4).

Analysis of the results by sex revealed that for men, there were no significant differences in intakes of total fat, mono- and polyunsaturated fats, omega 3 fatty acids, sugar, and alcohol, in addition to borderline non-significant differences for carbohydrates and saturated fats (Appendix Table A). Significant differences between the first and second administrations of the WebFFQ were found in men for intakes of total energy (0.9 MJ/day, *p* = 0.01), protein (13 g/day, *p* = 0.001), and fiber (3 g/day, *p* = 0.04), in addition to carbohydrates (20 g/day, *p* = 0.05) and saturated fats (3 g/day, *p* = 0.05). Correlations between absolute intakes of energy and macronutrients ranged from 0.73 for sugar to 0.87 for alcohol (all correlations significant at 0.01 level) (Appendix Table A).

In women, there were no differences for total fat, fatty acids, sugar, fiber, and alcohol, whereas significant differences were found between estimates from WebFFQ1 and WebFFQ2 for the intakes of energy (0.7 MJ/day, *p* = 0.02), protein (8 g/day, *p* < 0.01), and carbohydrates (18 g/day, 0.02). The correlations ranged from 0.62 for the intake of fiber to 0.91 for the intake of alcohol (all correlations significant at 0.01 level) (Appendix Table B).

Estimated proportions of energy from energy-providing nutrients were not significantly different in WebFFQ1 and WebFFQ2 (Table 4). The correlations found for E% estimates ranged from 0.75 for E% from protein and sugar to 0.88 for E% from alcohol (all correlations significant at 0.01 level) (Table 4). Correct cross-classification of participants with regard to E% ranged from 47% for protein to 67% for alcohol. Misclassification into the opposite quartile of E% was between 0 and 2% (Table 4). When analyzing the sexes separately, there were no significant differences between E% from WebFFQ1 and WebFFQ2 (Appendix Table A and B).

### Intakes of vitamins and minerals

Estimated absolute intakes of vitamins and minerals for all participants are presented in Table 5. There were differences in intakes of vitamins between the first and second administrations of WebFFQ for six out of eight vitamins, *p*-values ranging from <0.001 to 0.03. The intakes of vitamins A and E showed no significant differences, *p* = 0.3 and *p* = 0.2, respectively (Table 5). There were significantly different estimates for the absolute intakes of minerals between WebFFQ1 and WebFFQ2. The correlations between intakes from WebFFQ1 and WebFFQ2 ranged from 0.61 for copper to 0.82 for vitamin E (all correlations significant at 0.01 level) (Table 5). Eleven of 17 vitamins and minerals showed high correlation (≥0.7), and the rest showed correlations from 0.61 to 0.69. Analysis of cross-classification of all participants for intakes of vitamins and minerals showed that exact classification ranged from 48% for vitamin C and thiamine to 63% for vitamin E. Misclassification into the opposite quartile ranged from 0% for vitamins A and D to 5% for copper (Table 5).

**Table 5:** Estimated intake of vitamins and minerals, from the first (WebFFQ1) and second (WebFFQ2) administration of the WebFFQ, in The Reproducibility Study, n=164

Absolute intakes	webFFQ1		webFFQ2		p^a^	Difference^b^		r^c^	Cross-classification		
	median	IQR	median	IQR		Mean	95%CI		Exact	Exact + adjacent	Miclass^d^
Vitamin A, RAE/d	1477	984, 2812	1499	934, 2628	0.307	83	-58, 223	0.77	52	92	0
Vitamin C, mg/d	186	124, 264	175	114, 241	0.013	12.9	-1.3, 27.1	0.74	48	93	1
Vitamin D, μg/d	13.7	7.6, 29.7	12.6	7.2, 28.6	0.029	1.0	-2.1, 4.0	0.79	59	92	0
Vitamin E, mg/d	23.5	13.9, 38.8	20.5	13.6, 44.0	0.239	0.8	-2.4, 3.9	0.82	63	93	1
Niacin, mg/d	30.0	22.6, 42.3	27.5	19.9, 37.9	0.001	2,1	-0.4, 4.6	0.69	57	88	1
Vitamin B6, mg/d	2.43	1.83, 3.39	2.23	1.57, 3.06	0.010	0.1	-0.1, 0.4	0.70	60	89	2
Vitamin B12, μg/d	7.95	6.03, 11.70	7.50	5.63, 10.48	< 0.001	0.9	0.3, 1.4	0.74	53	89	1
Folate, μg/d	415	322, 543	379	276, 517	0.017	22.4	-6.6, 51.4	0.69	57	89	1
Calcium, mg/d	1005	761, 1305	946	686, 1179	0.008	77.0	15, 38	0.71	56	91	2
Iron, mg/d	13.5	10.1, 18.7	12.3	9.3, 16.8	0.004	1.6	-1.5, 4.7	0.62	53	86	2
Sodium, g/d	2.61	2.01, 3.48	2.38	1.89, 3.27	< 0.001	0.2	0.1, 0.3	0.74	53	93	2
Potassium, g/d	5.2	4.2, 6.4	4.7	3.8, 6.1	< 0.001	0.37	0.17, 0.57	0.77	58	93	1
Magnesium, mg/d	483	365, 584	435	332, 536	0.001	39.0	17, 60	0.70	50	92	4
Zinc, mg/d	14.2	11.0, 21.0	13.2	10.0, 18.2	0.007	1.3	0.0, 2.9	0.62	56	89	4
Selenium, μg/d	70.0	51, 98	62	46,0, 88.8	< 0.001	8.2	2.3, 14.1	0.67	50	88	3
Copper, mg/d	1.58	1.19, 2.54	1.48	1.03, 2.09	0.003	0.1	-0.1, 0.3	0.61	58	89	5
Phosphorus, g/d	1.9	1.6, 2.5	1.8	1.4, 2.4	0.001	0.16	0.08, 0.25	0.75	58	93	2

a p-value, test of difference in intake between first and second WebFFQ, Wilcoxon sign rank test. Significant difference set at p< 0.05.

b Absolute difference in intake between first and second WebFFQ, WebFFQ1-WebFFQ2.

c Spearman correlation between first and second WebFFQ.

d Misclassification of intake defined as opposite quartiles. IQR, Inter quartile range; FFQ, Food frequency questionnaire; mg/d, milligram per day; μg/d, microgram per day; 95% CI, 95% confidence interval; Misclass, classified into opposite quartile; RAE, retinol activity equivalents.

### Participant incentives

Based on the results from the feasibility study, we included different incentives for the participants in the reproducibility study. Of those who filled in the WebFFQ twice, 25% wanted written dietary feedback, 13% wanted a gift certificate, 55% wanted both dietary feedback and a gift certificate, and 7% did not want any of the incentives on offer.

## Discussion

We conducted two studies to assess the feasibility and reproducibility of a newly developed, online semi-quantitative FFQ.

### Feasibility study

According to the feasibility study, most participants found the WebFFQ easy to fill in, and the average time taken of ~40 min was as expected from earlier pilot study tests. The problems reported by the participants with regard to the WebFFQ included estimation of portion sizes, intakes of food items that varied by season, the length of the questionnaire, and the lack of alternative food items. With regard to portion size, our study showed that some participants found food portion photographs to be helpful in estimating portion sizes. Several web-based questionnaires have included food portion photographs to assist with estimations of portion size (14–17). Visual aids for estimating portion sizes have shown to be favored by participants in studies exploring different portion-size estimation aids (18), and evaluation studies have shown that participants think that the pictures help them in estimating portion sizes (14, 15). Due to the small participant sample, results from the focus group interviews were interpreted with caution and used as guiding information in the planning of the reproducibility study. Future developments of the WebFFQ should include optimized portion-size pictures (i.e. using more informative pictures with examples of household measures), revised questions about seasonal foods, and a revised list of available food items to comply more fully with changing food trends and food habits in the Norwegian population at large.

### Participation rate

The participation rate was low for both studies. This was not unexpected, given that low participation rates were found in earlier studies, in which participants were recruited from the general population for evaluation studies (12, 19, 20). Levels of non-participation seem also to have increased in epidemiological studies in recent decades (21–23). At the time, the WebFFQ had no way of saving partly filled-in questionnaires. With a long questionnaire like the WebFFQ and without a technical solution for saving registrations halfway through, we speculate that some potential participants might have started but not completed the registration, and therefore not been included in the study, adding to the high rate of non-participation. It is hoped that further technical developments will resolve this issue. Other reasons for non-participation may have included the following: a general increase in studies requesting participants; a general decrease in volunteerism in western countries; studies must give something back to participants in exchange for their time and effort to make it worth their while; and last but not least, scientific studies may have become increasingly demanding for participants (21). Additionally, factors such as age, sex, ethnicity, education level, employments status, socioeconomic status, and smoking status may have influenced the participation rate (21, 23). The motivation to undertake the work and give up time required by such studies poses a challenge to the way we design methodological studies. By offering incentives to those participating in the reproducibility study, including a monetary gift certificate, we had hoped to increase the participation rate, as seen in other studies (24–26), and 93% of the participants did choose to receive one or more of the incentives. However, even with these incentives, the participation rate was still rather low.

In both studies, the samples consisted of a higher proportion of people aged 45–66 years compared to the general population of Norway. The study populations also had fewer men, fewer male smokers, and a higher proportion of people with a high level of education compared with the general population (27). The characteristics of the study sample probably affected the results, making them less representative for the general population. A study sample more in line with the general population may have had different outcomes.

### Reproducibility study

Our results suggest that the WebFFQ is able to reproduce intakes of food, energy, and nutrients at group level. A few systematic differences between the estimates from the two WebFFQ administrations were observed. These were small compared with the average daily intake. Additionally, based on the correlations and classification agreement tests, we found that the WebFFQ was able to reproduce the ranking of participants adequately.

The estimated intakes of most food categories did not differ between the two administrations of the WebFFQ at group level, and most correlations were high (≥0.7). The correlations found in our study were in the same range or higher than those presented in other reproducibility studies of online FFQs (17, 28). However, for most food groups, the absolute intakes showed a tendency to decrease in WebFFQ2, and important food categories including potatoes, fruit, meat, fish, and milk all showed lower estimated intakes at the second administration. A shift toward lower intakes in the second administration of FFQs has also been reported in other studies (29–32). The two administrations of the WebFFQ took place during winter and spring, respectively. Natural variation over time with regard to diet and use and availability of varieties of different foods, especially vegetables, fruit, and berries, presents a challenge to participants when registering their average habitual intake over a year. The observed differences may also have resulted from measurement errors inherent in the FFQ methodology (2, 33). FFQs with long lists of food items have been shown to overestimate food intake (34). We speculate that from what they may have learned during the first administration of the WebFFQ, participants may have been less prone to overestimate food intake at the second administration. However, although lower estimates were obtained from the second administration, the median differences at group level were small and within an average portion size for the respective food groups.

Total energy intakes in the present reproducibility study were high, and using the Goldberg evaluation of energy intake (35) indicates overreporting. It is also higher than the average total energy intake found in the large population-based Tromsø Study 2015–16 (9.7 MJ/day), which used the paper version of the WebFFQ (36). Still, in a validation study of the WebFFQ among women and using double label water as the reference, no significant difference in energy intake was observed between the WebFFQ and the reference method (8). The small study sample in our study may contribute to the results, and we cannot rule out a possible overestimation of food intake in the present study. The changes in food intakes from the first to second administration of the WebFFQ affected the estimated intakes of energy and some of the nutrient intakes. Total energy intake decreased in the second administration, in agreement with earlier studies (28, 29). However, E% estimations did not differ between the first and second administrations of the WebFFQ. This is in agreement with an earlier FFQ test–retest study in a Norwegian population, which found reduced intakes of energy and most nutrients but no difference in E% (29). The percentages of participants classified into exact plus adjacent food categories were consistently high, ranging from 86% for bread to 97% for coffee. This is comparable to the results for the online Food4Me FFQ, in which classification percentages were in the same range (28).

The participation rate in the reproducibility study was low, which was a limitation to the study. Results from a biased study sample may not be representative of a wider national population. Additionally, the number of men in the reproducibility study was low, which may have influenced the correlation analyses in men. One strength of the reproducibility study was the long time between the first and second administrations of the WebFFQ, which limited the learning effect (11).

In an earlier validation study, we evaluated the WebFFQ with regard to intakes of nutrients and food groups using doubly labeled water and 24-h recall (8). At group level, the WebFFQ was evaluated to estimate adequately the absolute intakes of macronutrients and foods groups and to rank individuals adequately according to intakes of nutrients and food groups. Further evaluation using biomarkers of intake may be warranted to evaluate estimates of specific nutrients in more detail.

## Conclusion

The self-administered online WebFFQ demonstrates good feasibility and reproducibility for estimations of food groups, energy, and nutrients at group level. Therefore, together with the results of the earlier validation study, the WebFFQ may be considered suitable for dietary assessments in healthy adults in the Norwegian population.

## Figures and Tables

**Appendix Figure 1A:**
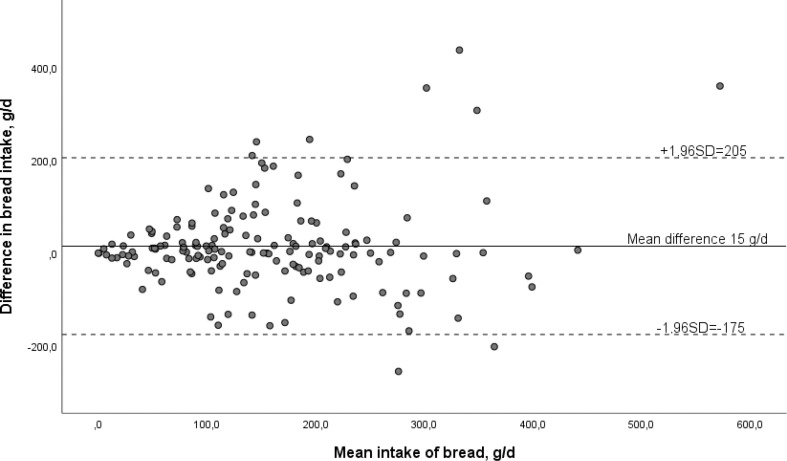
Bland-Altman plot of the intake of bread from WebFFQ1 and WebFFQ2. Mean intake on the x-axis against the difference in intake (WebFFQ1-WebFFQ2) on the y-axis, in grams per day.

**Appendix Figure 1B:**
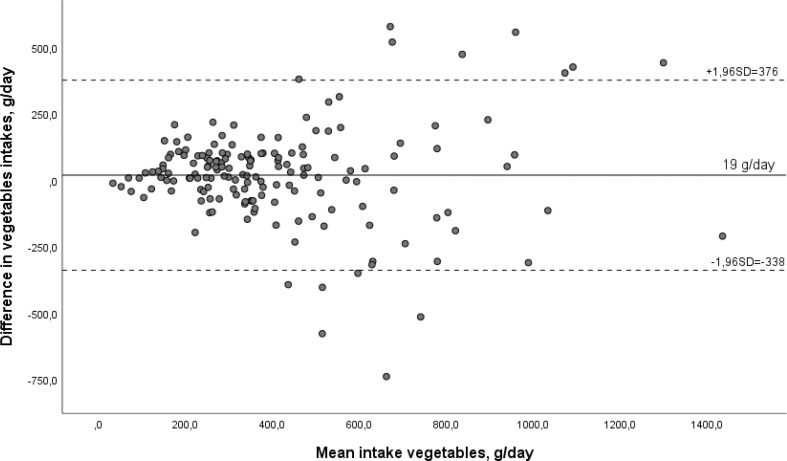
Bland-Altman plot of the intake of vegetables from WebFFQ1 and WebFFQ2. Mean intake on the x-axis against the difference in intake (WebFFQ1-WebFFQ2) on the y-axis, in grams per day.

**Appendix Figure 1C:**
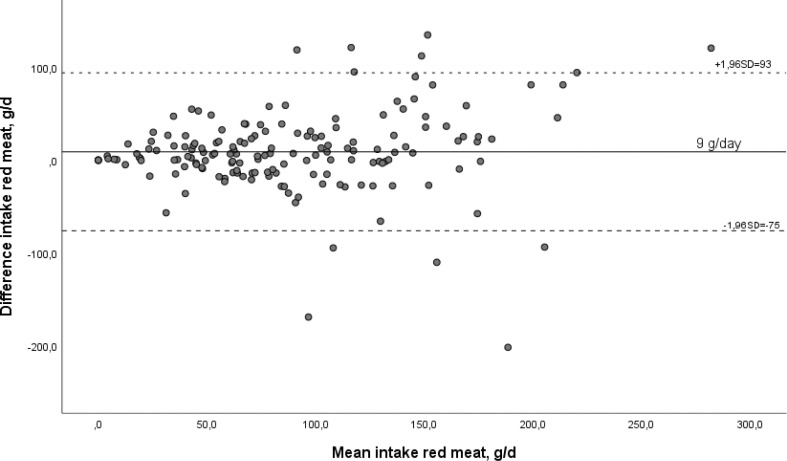
Bland-Altman plot of the intake of red meat from WebFFQ1 and WebFFQ2. Mean intake on the x-axis against the difference in intake (WebFFQ1-WebFFQ2) on the y-axis, in grams per day.

**Appendix Figure 1D:**
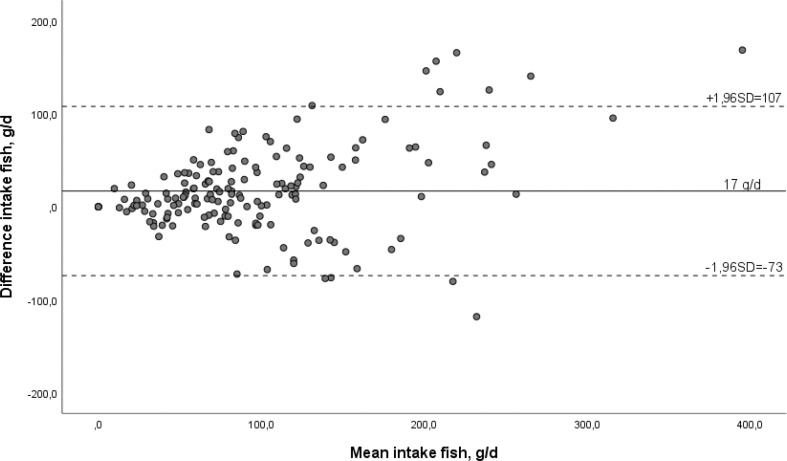
Bland-Altman plot of the intake of fish from WebFFQ1 and WebFFQ2. Mean intake on the x-axis against the difference in intake (WebFFQ1-WebFFQ2) on the y-axis, in grams per day.

## Appendix

**Appendix Table A:** Estimated intake of macronutrients in men, from the first (WebFFQ1) and second (WebFFQ2) administration of the WebFFQ, in The Reproducibility Study, n=68

Absolute intakes	WebFFQ1		WebFFQ2		p^a^	Difference^b^		r^c^
	Mean	95% CI	Mean	95% CI		Mean	95% CI	
Energy, MJ/day	12.2	11.1, 13.3	11.3	10.3, 12.2	0.012	0.9	0.2, 1.7	0.74
Protein, g/day	127	115, 139	114	104, 123	0.001	13	6, 21	0.74
Fat, g/day	123	109, 136	114	101, 127	0.052	9	0, 18	0.79
Saturated fat, g/day	40	36, 44	37	33, 41	0.048	3	0, 6	0.75
Mono-unsat, fat, g/day	47	42, 53	44	39, 49	0.069	3	0, 7	0.79
Poly-unsat, fat, g/day	25	21, 28	23	20, 27	0.155	2	-1, 4	0.83
Omega 3, g/day	8	6, 10	8	6, 9	0.331	0	0, 1	0.84
Carbohydrates, g/day	294	266, 322	274	247, 301	0.045	20	0, 40	0.77
Sugar, g/day	33	27, 39	32	27, 38	0.615	1	-3, 5	0.73
Fiber, g/day	38	34, 43	35	31, 40	0.035	3	0, 6	0.79
Alcohol, g/day^d^	6	2 to 11	7	5 to 8	0.101	1	0, 3	0.87
**Energy percent, E%**								
Protein	17.8	17.1, 18.5	16.7	15.4, 17.9	0.057	1.1	-0.03, 2.3	0.40
Fat	36.5	34.4, 38.6	35.3	31.7, 39.0	0.430	1.2	-1.8, 4.1	0.58
Carbohydrates	41.1	39.1, 43.1	40.2	36.0, 44.5	0.629	0.9	-0.2, 0.3	0.50
Sugar	4.5	3.9, 5.2	4.8	3.8, 5.9	0.447	-0.3	-1.1, 0.5	0.63
Fiber	2.5	2.3, 2.7	2.5	2.1, 2.8	0.770	0.04	-0.2, 0.3	0.69
Alcohol	2.0	1.5, 2.6	1.8	1.3, 2.3	0.247	0.2	-0.2, 0.6	0.70

a p-value, test of difference in intake between first and second WebFFQ, Paired sample t-test. Significant difference set at p < 0.05.

b Absolute difference in intakes from WebFFQ1 and WebFFQ2.

c Pearson correlation between first and second WebFFQ.

d Skewed distribution, data and analyses presented as median, Inter quartile range, Wilcoxon signed rank test and Spearman correlation. FFQ, Food frequency questionnaire; g/day, gram per day; 95% CI, 95% confidence interval.

**Appendix Table B:** Estimated intake of macronutrients in women, from the first (WebFFQ1) and second (WebFFQ2) administration of the WebFFQ, in The Reproducibility Study, n=96

Absolute intakes	WebFFQ1		WebFFQ2		p^a^	Difference^b^		r^c^
	Mean	95% CI	Mean	95% CI		Mean	95% CI	
**Energy, MJ/day**	**10.1**	**9.4, 10.9**	**9.5**	**8.8, 10.2**	**0.02**	**0.7**	**0.3, 1.3**	**0.70**
**Protein, g/day**	**105**	**97, 113**	**97**	**89, 104**	**<0.01**	**8**	**3, 14**	**0.71**
**Fat, g/day**	**104**	**94, 114**	**98**	**89, 107**	**0.10**	**6**	**-1, 13**	**0.75**
**Saturated fat, g/day**	**35**	**31, 38**	**32**	**30, 35**	**0.15**	**2**	**-1, 5**	**0.74**
**Mono-unsat, fat, g/day**	**40**	**36, 43**	**37**	**33, 41**	**0.14**	**2**	**-1, 5**	**0.77**
**Poly-unsat, fat, g/day**	**21**	**18, 23**	**19**	**17, 22**	**0.12**	**1**	**0, 3**	**0.80**
**Omega 3, g/day**	**6**	**5, 7**	**6**	**5, 7**	**0.10**	**0**	**0, 1**	**0.82**
**Carbohydrates, g/day**	**241**	**220, 261**	**223**	**204, 242**	**0.02**	**18**	**3, 32**	**0.66**
**Sugar, g/day**	**33**	**27, 39**	**28**	**24, 32**	**0.08**	**4**	**0, 9**	**0.78**
**Fiber, g/day**	**34**	**31, 37**	**32**	**29, 35**	**0.83**	**2**	**0, 4**	**0.62**
**Alcohol, g/day** ^d^	**4**	**1 to 7**	**5**	**4 to 7**	**0.45**	**1**	**-1 to 2**	**0.91**
**Energy percent, E%**								
**Protein**	**18.0**	**17.3, 18.6**	**17.1**	**16.0, 18.3**	**0.09**	**0.8**	**-0.1, 1.8**	**0.55**
**Fat**	**37.3**	**35.7, 38.9**	**36.8**	**33.8, 39.8**	**0.73**	**0.5**	**-2.2, 3.2**	**0.45**
**Carbohydrates**	**40.1**	**38.4, 41.8**	**39.0**	**36.3, 41.5**	**0.34**	**1.2**	**-1.3, 3.8**	**0.35**
**Sugar**	**5.2**	**4.5, 5.9**	**4.8**	**4.2, 5.5**	**0.22**	**0.4**	**-0.2, 1.0**	**0.59**
**Fiber**	**2.7**	**2.6, 2.9**	**2.7**	**2.5, 2.9**	**0.66**	**0.04**	**-0.1, 0.2**	**0.53**
**Alcohol**	**1.9**	**1.4, 2.3**	**1.9**	**1.4, 2.4**	**0.97**	**0.0**	**-0.3, 0.3**	**0.80**

a p-value, test of difference in intake between first and second WebFFQ, Paired sample t-test. Significant difference set at p < 0.05.

b Absolute difference in intakes from WebFFQ1 and WebFFQ2.

c Pearson correlation between first and second WebFFQ.

d Skewed distribution, data and analyses presented as median, Inter quartile range, Wilcoxon signed rank test and Spearman correlation. FFQ, Food frequency questionnaire; g/day, gram per day; 95% CI, 95% confidence interval.

**Appendix Table C:** Estimated intake of vitamins and minerals per 10 MJ, from the first (WebFFQ1) and second (WebFFQ2) administration of the WebFFQ, in The Reproducibility Study, in all participants, n=164

Intakes pr 10 MJ	webFFQ1		webFFQ2		p^a^	Difference^b^		r^c^	Cross-classification		
	Mean	95% CI	Mean	95% CI		Mean	95% CI		Exact	Exact + adjacent	Miclass^d^
Vitamin A, RAE/10MJ	1840	1.7, 2.0	1934	1740, 2129	0.2	-94.5	-223.7, 34.5	0.76	56	92	2
Vitamin C, mg/10MJ	203	186, 221	204	185, 223	0.9	-0.79	-15.9, 14.3	0.66	44	90	2
Vitamin D, μg/10MJ	30.2	23.8, 36.5	31.2	24.4, 37.9	0.4	-1.00	-3.44, 1.44	0.93	59	92	1
Vitamin E, mg/10MJ	37.2	31.1, 43.3	38.9	32.3, 45.4	0.2	-1.74	-4.36, 0.88	0.92	63	93	0
Niacin, mg/10MJ	32.6	30.1, 35.1	33.5	29.9, 37.0	0.6	-0.87	-3.97, 2.22	0.53	61	91	3
Vitamin B6, mg/10MJ	2.8	2.5, 3.0	2.9	2.5, 3.3	0.4	-0.13	-0.46, 0.21	0.51	60	91	3
Vitamin B12, μg/10MJ	8.6	8.1, 9.1	8.5	8.0, 8.9	0.5	0.15	-0.27, 0.56	0.59	41	86	3
Folate, μg/10MJ	435	407, 464	451	409, 493	0.4	-15.9	-49.8, 18.1	0.63	56	93	1
Iron, mg/10MJ	13.8	13.1, 14.5	15.4	13.2, 17.5	0.1	-1.59	-3.51, 0.33	0.45	57	88	3
Na, g/10MJ	2.7	2.6, 2.8	2.6	2.5, 2.7	0.5	29.1	-62.8, 121.0	0.63	54	95	2

a p-value, test of difference in intake between first and second WebFFQ, Paired sample t-test. Significant difference set at p < 0.05.

b Absolute difference in intakes from WebFFQ1 and WebFFQ2.

c Pearson correlation between first and second WebFFQ.

d Misclassification of intake defined as opposite quartiles. FFQ, Food frequency questionnaire; 95% CI, 95% confidence interval; mg/d, milligram per day; μg/d, microgram per day; MJ, mega joule.
